# DARS2 Promotes Bladder Cancer Progression by Enhancing PINK1-Mediated Mitophagy

**DOI:** 10.7150/ijbs.107632

**Published:** 2025-01-27

**Authors:** Dongqing Li, Hang Su, Xiaolin Deng, Yuan Huang, Zihuan Wang, Jinge Zhang, Chen Chen, Zaosong Zheng, Qiong Wang, Shanchao Zhao, Zhe-Sheng Chen, Haiyong Chen, Lina Hou, Wanlong Tan, Fei Li

**Affiliations:** 1 Department of Urology, Nanfang Hospital, Southern Medical University, Guangzhou, Guangdong 510515, P.R. China.; 2 Department of Urology, Ganzhou People's Hospital, Ganzhou, P.R. China.; 3 Department of Urology, the Fifth Affiliated Hospital of Southern Medical University, Guangzhou, Guangdong 510500, P.R. China.; 4 Department of Pharmaceutical Sciences, College of Pharmacy and Health Sciences, St. John's University, Queens, New York, 11439, USA.; 5 School of Chinese Medicine, LKS Faculty of Medicine, The University of Hong Kong R619, 3 Sassoon Road, Pokfulam, Hong Kong SAR, China.; 6 Department of Huiqiao Medical Center, Nanfang Hospital, Southern Medical University, Guangzhou, Guangdong 510515, P.R. China.

**Keywords:** Bladder cancer, DARS2, PINK1, Mitophagy, Cellular senescence

## Abstract

Globally, bladder cancer is the tenth most common cancer. Mitophagy, a critical process regulating mitochondrial quantity and quality, has attracted increasing attention for its pivotal function in cancer. Nonetheless, its roles and underlying mechanisms in bladder cancer are yet to be elucidated. Therefore, in this study, 16 mitophagy-related genes were screened to construct a robust prognostic model with exceptional predictive accuracy for the outcomes of patients with bladder cancer. Of these genes, DARS2 was identified as a key regulator that significantly affected cancer progression. The findings established that DARS2 promoted the G1-to-S phase transition by upregulating CDK4 expression, thereby suppressing cellular senescence and driving cell proliferation. In addition, DARS2 augmented PINK1 expression, leading to increased PINK1-mediated mitophagy. Both *in vitro* and *in vivo* experiments confirmed that DARS2 inhibited cellular senescence and facilitated tumor progression by enhancing PINK1-mediated mitophagy. The observations from this study have provided novel insights into the multifaceted roles of DARS2-mediated mitophagy in bladder cancer. Targeting DARS2 and its regulation of mitophagy is a promising therapeutic strategy to improve the outcomes for patients with bladder cancer.

## 1. Introduction

Bladder cancer (BCa) is one of the most common malignancies of the urinary system and is the fourth most prevalent cancer in men^1^. Worldwide, approximately 573,000 new cases and 213,000 deaths are reported annually, making it a substantial public health concern^1^, ^2^. The malignant progression of BCa is driven by complex mechanisms, including genetic mutations, epigenetic alterations, and the tumor microenvironment^3^-^5^. Comprehending the biological drivers of BCa progression is therefore critical for identifying novel therapeutic strategies.

Cellular senescence, an irreversible state of cell cycle arrest, is a hallmark of cancer^6^, ^7^. This process is characterized by the activation of DNA damage response, resistance to apoptosis, inhibition of cyclin-dependent kinases, and secretion of proinflammatory factors termed senescence-associated secretory phenotypes (SASPs)^8^, ^9^. Although senescence has conventionally been perceived as a tumor-suppressive mechanism, its role in cancer is more complex^10^. Senescence exerts a dual effect in cancer as it suppresses tumor formation by halting proliferation and also promotes tumor progression via SASP-mediated angiogenesis and tissue remodeling^11^-^13^. This dual role highlights the need for strategies that modulate senescence to control BCa progression.

Mitophagy, the primary mechanism governing mitochondrial quality control, has been implicated in both cellular senescence and tumor progression^14^-^17^. Dysfunctional mitochondria are eliminated via mitophagy, which reduces reactive oxygen species (ROS) and mitochondrial DNA mutations, thereby delaying senescence and maintaining cellular homeostasis^18^, ^19^. Given its role in regulating cellular energy and stress responses, mitophagy is emerging as a critical process in cancer biology. The PINK1/Parkin pathway is a central regulator of mitophagy and has been extensively studied^20^. In BCa, rapamycin-induced AMPK activation has been reported to enhance mitophagy, suppressing cell proliferation and migration^21^. Similarly, MCM7 has been observed to upregulate mitophagy and reduce p53 accumulation, maintaining BCa cell stemness^22^. Nevertheless, the precise role of mitophagy in BCa progression is poorly understood and warrants further investigation. DARS2 (aspartyl-tRNA synthetase 2) is a key mitochondrial aminoacyl-tRNA synthetase whose dysfunction can impair mitochondrial protein synthesis, resulting in mitochondrial dysfunction^23^. It is reported that DARS2 plays a pivotal regulatory role in the progression and apoptosis of hepatocellular carcinoma and lung adenocarcinoma^24^, ^25^. However, the effects of DARS2 on BCa and mitophagy remain unknown.

Therefore, in this study, 16 mitophagy-related genes were screened to construct a prognostic model, demonstrating its strong predictive value for prognosis in patients with BCa. Furthermore, DARS2, which was significantly upregulated in BCa, was identified as a key factor. Interestingly, DARS2 upregulated PINK1-mediated mitophagy by promoting PINK1 mitochondrial expression, thus inhibiting cellular senescence and promoting cell proliferation. These findings emphasize the intrinsic association among DARS2, mitophagy, and cellular senescence in BCa.

## 2. Materials and methods

### 2.1. Data collection and acquisition

A total of 824 MRGs (Supplementary Table 1) were obtained from the GeneCards database (https://www.genecards.org/) with a relevance score of >1. Prognosis-related genes (PRGs) in BCa were identified through Kaplan-Meier survival analysis and a univariate Cox proportional hazards model, with *p* < 0.05 considered to indicate statistical significance in TCGA and GSE13507. The R package VennDiagram (CRAN - Package VennDiagram (r-project.org)) was used to identify the overlapping MRGs and PRGs.

Transcriptomic and clinical data for BCa patients were sourced from TCGA, GSE13507, and IMvigor210. Single-cell transcriptomic profiles were retrieved from GSE211388 and analyzed using Seurat. Quality control was performed to eliminate cells based on the following exclusion criteria: (1) fewer than 200 or more than 4,000 genes, (2) fewer than 1,000 reads, (3) UMI counts outside the range of 500-15,000, or (4) >10% of mitochondrial UMIs. The data were normalized using LogNormalize, and the top 2,000 highly variable genes were identified. Dimensionality reduction was performed using PCA, t-SNE, and UMAP. Cell clustering was conducted using FindNeighbors and FindClusters, with cell annotations obtained via SingleR (Supplementary Table 3).

### 2.2. Construction of a prognostic MRG model

A prognostic model was constructed by using machine learning-integrated algorithms. Ultimately, a combination of the LASSO and SuperPC algorithms was selected as the final model.

The risk score was calculated based on the following formula:



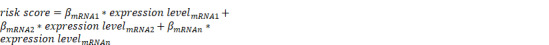



where 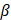 is the risk coefficient of each factor. The risk score for each patient was calculated as follows: Risk score = ANXA2 expression * 0.148207972739551 + CDK5RAP3 expression * (-0.0216587905197319) + CSNK2A2 expression * 0.00595383260568351 + DARS2 expression * 0.0231697095929072 + DCXR expression * 0.209058645007052 + FASN expression * 0.165701072850379 + GFPT2 expression * 0.125204197049992 + MAP1A expression * 0.109561386716842 + PRDX6 expression * 0.127684552011834 + SAR1A expression * 0.0211344182319381 + SCD expression * 0.00674879338858188 + SCO2 expression * (-0.423184153287598) + SPTBN2 expression * 0.106972468192409 + TFRC expression * 0.0712419818434375 + TRIM27 expression * (-0.239048049127721) + UBC expression * 0.1174018352924.

### 2.3. Sample sources

BCa samples were obtained from patients under treatment at the Department of Urology, Nanfang Hospital, Southern Medical University during March 1-31, 2023. Relevant clinical records and samples of all BCa cases were approved for use by the Ethics Committee. Before seeking their approval, all patients were provided with informed consent.

### 2.4. Cell culture and reagents

T24 and SW780 cells were utilized in this study. The experimental cells were maintained at 37°C in a humidified atmosphere containing 5% CO_2_ and cultured in DMEM basic medium (Gibco, C11995500BT) with 10-15% fetal bovine serum (164210, China). The carbonyl cyanide m-chlorophenyl hydrazone (CCCP) (MedChemExpress, HY-100941) was used to induce mitophagy.

### 2.5. RNA interference and lentiviral transfection

DARS2-targeting siRNA/shRNA was synthesized by GenePharma (Guangzhou, China). The overexpression plasmids for PINK1 were purchased from Miaoling Biotech (Wuhan, China). Transfection of siRNA was performed using Lipofectamine 3000 (Servicebio, USA) for 48 h, according to the manufacturer's protocol. For DARS2 silencing, lentiviral infection of T24 or SW780 cells was performed using lentivirus (Genechem, China), followed by the selection with DMEM containing 4 μg/mL puromycin (GBCBIO, J593-25mg) for 48-72 h. The siRNA/shRNA sequence for PINK1 and DARS2 is provided in the Supplementary Table 7.

### 2.6. Western blotting

Protein lysates were separated by SDS-PAGE and transferred onto PVDF membranes. These membranes were then incubated overnight at 4°C with primary antibodies against β-actin, DARS2, PINK1, and others. After incubation with secondary antibodies at room temperature for 1 h, protein detection was performed by using an enhanced chemiluminescence detection system. The following antibodies were used in this study: Anti-DARS2 (Proteintech, 13807-1-AP, 1:2000), Anti-p53 (Proteintech, 10442-1-AP, 1:2000), Anti-p21 (Proteintech, 10355-1-AP, 1:2000), Anti-CDK6 (Proteintech, 14052-1-AP, 1:2000), Anti-CDK4 (Proteintech, 11026-1-AP, 1:2000), Anti-LC3 (Proteintech, 14600-1-AP, 1:2000), Anti-PINK1 (Proteintech, 23274-1-AP, 1:2000), and Anti-Parkin (Proteintech, 14060-1-AP, 1:2000). β-actin (Proteintech, 20536-1-AP, 1:2000) was used as the loading control. Protein expression was quantified using ImageJ software.

### 2.7. Cellular senescence staining

Cellular senescence was tested by using the SA-β-gal kit (KeyGEN BioTECH, KGA5101-100) as per the manufacturer's instructions. T24 and SW780 cells were fixed at 26°C for 16 min and cultured overnight with the SA-β-gal solution in a CO_2_-free incubator at 37°C. Then, the cells were observed under a conventional optical microscope. Image data were analyzed quantitatively by using ImageJ software.

### 2.8. Cell viability assay

The viability of T24 and SW780 cells was assessed by the CCK8 assay. We seeded 3 × 10^3^ cells/well in a 96-well plate. After attachment, the cells received the indicated treatments before the CCK8 reagent was added. Then, the cells were cultured for approximately 2 h in humidified incubators at 37°C with 5% CO_2_. We then measured the optical density at 450 nm (OD450) by using an ELISA reader.

### 2.9. Flow cytometry

Cell cycle analysis was performed in accordance with the manufacturer's protocol by using the cell cycle detection kit (KeyGEN BioTECH, KGA9101-100). Briefly, the cells were fixed in 70% cold ethanol (500 μL) overnight and stained with PI/RNase A staining solution (500 μL). Next, the cells were incubated for 30-60 min in the dark. The ROS levels were measured by using a ROS detection kit (S0033M, Beyotime) after 30 min of incubation. The mitochondrial mass and membrane potential were evaluated by using Mitotracker-green (C1048, Beyotime) and TMRE (C2001S, Beyotime), respectively, at a concentration of 10 nM. A BD Accuri C6 flow cytometer together with FlowJo X software was used to collect and analyze the data.

### 2.10. Immunofluorescence

T24 and SW780 cells were incubated with 15 nM Mitotracker-green (C1048, Beyotime) and 15 nM Lysotracker-red (C1046, Beyotime) for 25 min, with the nuclei stained using Hoechst 33342 (C1025, Beyotime). Live-cell fluorescence images were captured using either a laser confocal microscope (FV3000) or a super-resolution microscope (N-SIM+N-STORM, Nikon). In addition, after transfection of the cells with mt-Keima plasmids for 3 days and nuclei stained using Hoechst 33342, live-cell figures were taken through a laser confocal microscope. Image analysis was performed using Imaris software (Oxford Instruments, UK).

### 2.11. Immunohistochemistry (IHC) staining

The tissue samples from BCa patients and nude mice were subjected to standard IHC procedures (fixed in formalin, embedded in paraffin, and sectioned). The slides were incubated overnight at 4°C with primary antibodies (anti-DARS2 1:200, anti-PINK1 1:200, anti-CDK4 1:200), followed by incubation with secondary antibodies (1:250) for 40 min at 25°C. These slides were developed using DAB and counterstained with hematoxylin. The images were captured by using a slide scanner (DX300, HISTECH, China).

### 2.12. Animal experiments

This project was approved by the Ethics Committee of Nanfang Hospital, Southern Medical University. T24 cells (1 × 10^7^ cells/mouse) transfected with sh-DARS2 or oe-PINK1 were subcutaneously injected into BALB/c nude mice (4-week-old, female). Tumor volume and mice body weight were recorded daily starting 14 days post-injection. The nude mice were euthanized on day 28. Their tumor tissues were isolated, photographed, weighed, and subjected to immunohistochemical staining and histopathological evaluation.

### 2.13. Statistical analysis

All statistical analyses and data visualizations were performed by using the GraphPad Prism 8 software. Statistical significance was determined by one-way ANOVA or Student's t-test, with results presented as the mean ±SEM from at least three independent experiments (*p < 0.05, **p < 0.01, ***p < 0.001, ****p < 0.0001, ns: not significant).

## 3. Results

### 3.1. The prognostic model based on mitophagy-related genes could effectively predict prognosis in BCa

**Figure 1:**
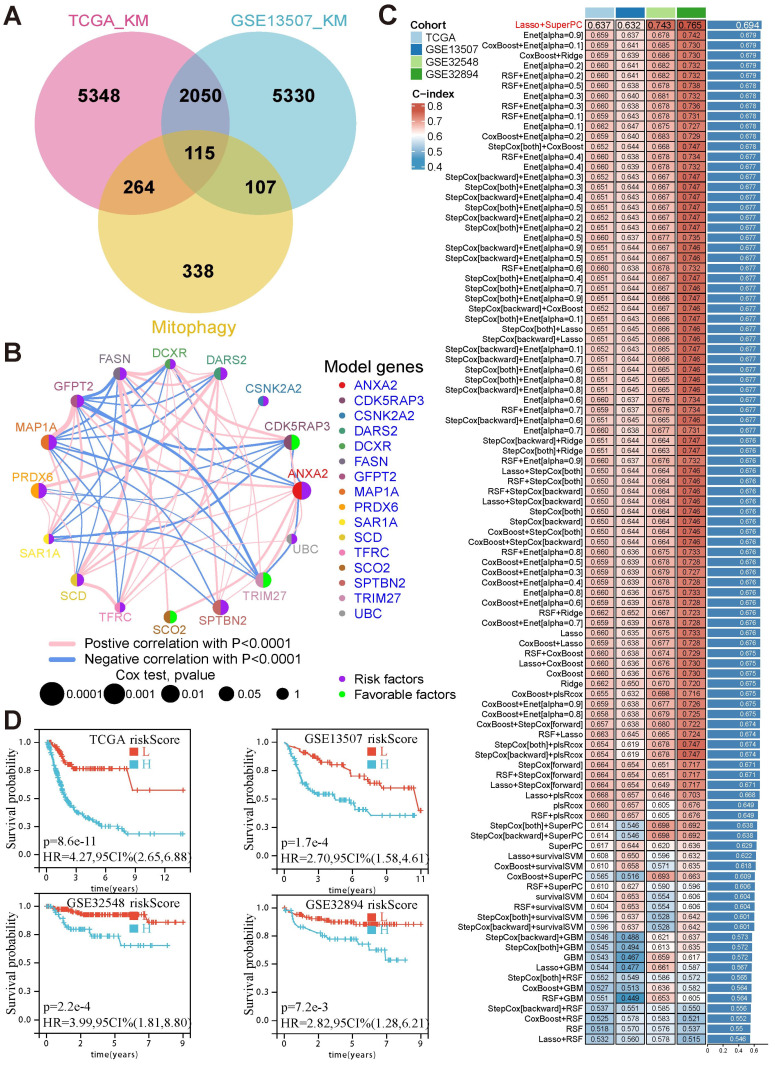
** The prognostic model based on mitophagy-related genes could effectively predict prognosis in BCa.** (A) Venn diagram depicting the intersection between prognosis-related genes from TCGA and GSE13507 cohorts and MRGs, yielding 115 candidate genes. (B) The co-expression network of the 16 model genes, with node size representing p-values from Cox analysis and edge thickness representing the strength of correlation. (C) Comparison of the C-index values across four cohorts using different machine-learning algorithms, with the LASSO and SuperPC combination demonstrating the highest average performance. (D) Kaplan—Meier survival curves illustrating significant survival differences between the high- and low-risk groups across TCGA, GSE13507, GSE32548, and GSE32894 cohorts.

To comprehensively examine the role of mitophagy in BCa, 824 mitophagy-related genes (MRGs) were collected (Supplementary Table 1). A total of 115 candidate genes were identified from the intersection of MRGs and prognosis-related genes (Fig. 1A). Based on these candidates, machine learning algorithms were assessed to construct a prognostic model by identifying 16 hub genes that were closely related to each other (Fig. 1B, Supplementary Table 2). Finally, the combination of LASSO and SuperPC algorithms was selected owing to its superior predictive performance, achieving the highest average C-index across four cohorts (TCGA: 0.637, GSE13507: 0.632, GSE32548: 0.743, GSE32894: 0.765) (Fig. 1C). Of the 16 model genes, CDK5RAP3, TRIM27, and SCO2 were determined to be favorable prognostic factors, whereas the remaining 13 genes, including DARS2, ANXA2, and PRDX6, were risk factors.

Risk scores were calculated for each patient based on the weighted expression of these 16 MRGs. Patients were stratified into high- and low-risk groups using the median risk score as the cutoff. Survival analysis revealed that patients in the high-risk group consistently exhibited poor outcomes across all cohorts (TCGA: HR = 4.27, *p* < 0.001; GSE13507: HR = 2.70, *p* < 0.001; GSE32548: HR = 3.99, *p* < 0.001; GSE32894: HR = 2.82, *p* = 0.0027) (Fig. 1D). These findings establish the critical role of these 16 MRGs in BCa prognosis and highlight their potential as prognostic biomarkers and therapeutic targets.

### 3.2. Cellular senescence could play a pivotal role in the progression of BCa

**Figure 2:**
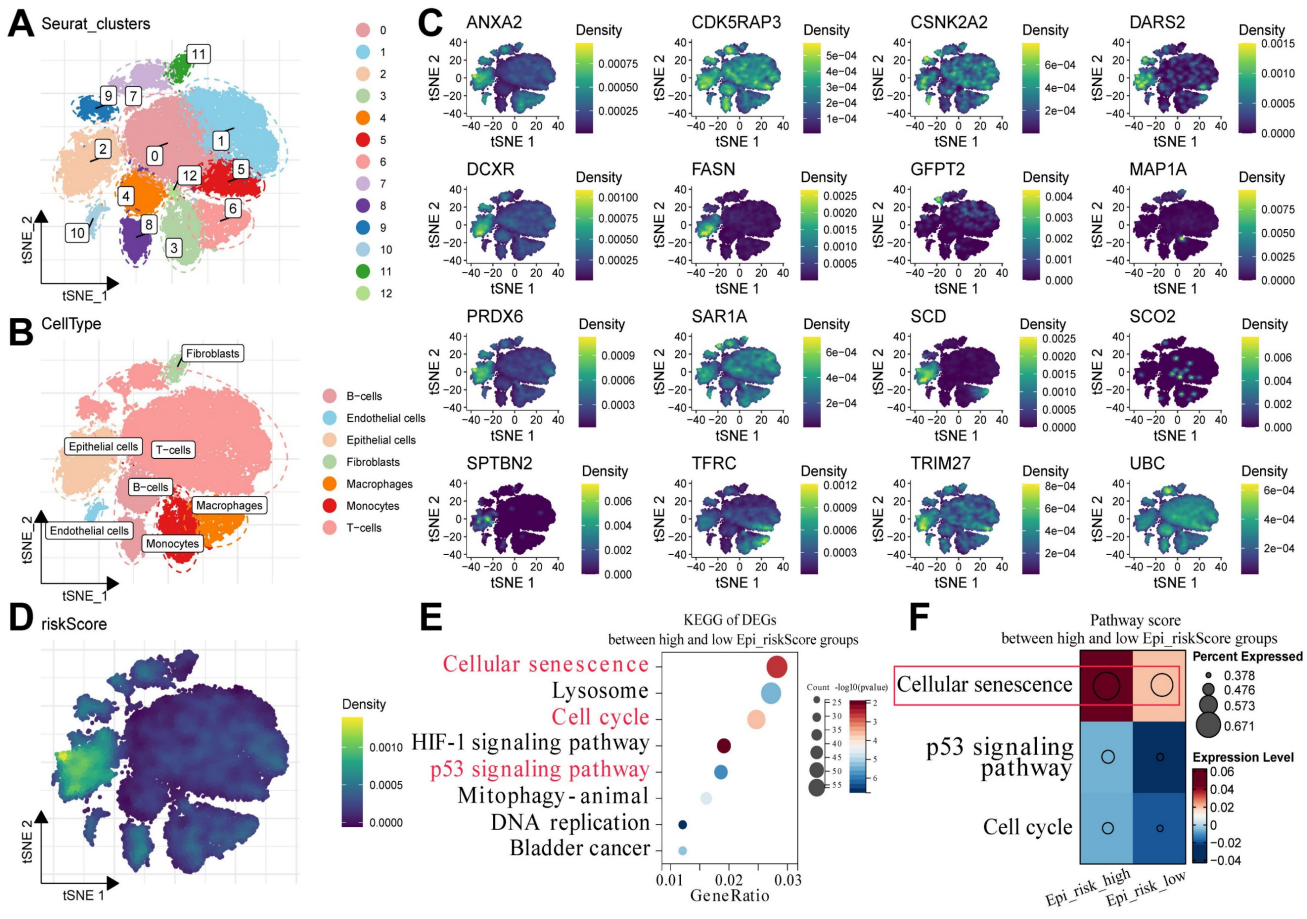
** Cellular senescence could play a pivotal role in the progression of BCa.** (A) The cells were clustered into Seurat_clusters, with distinct colors representing clusters and numbers indicating cluster IDs. (B) The cell types were distributed across tSNE space, with epithelial cells, fibroblasts, T-cells, and others, labeled by color. (C) The tSNE expression density maps displaying the distribution of the 16 prognostic model genes, with the density levels indicated by color. (D) Epi_riskScore distribution was visualized in the tSNE space, with colors indicating its density in epithelial cells. (E) KEGG enrichment analysis of DEGs between the high and low Epi_riskScore groups highlighted pathways such as cellular senescence, cell cycle, and p53 signaling. The X-axis indicated GeneRatio, with dot size representing gene counts and color reflected -log10(p-value). (F) Pathway activity scores between high and low Epi_riskScore groups were compared, with dot size representing the percentage of expressed genes and color indicating the expressions.

To determine the potential mechanisms underlying the survival differences between the high- and low-risk groups, the expression patterns of the 16 MRGs were detected at the single-cell level. The BCa single-cell dataset GSE211388 was collected, followed by dimensionality reduction clustering and annotation of cell subpopulations (Supplementary Table 3). In addition, 13 clusters were identified, along with several major cell types, such as epithelial cells, fibroblasts, T cells, and B cells (Fig. 2A, B). Subsequently, the 16 MRGs were projected onto the single-cell atlas, which showed that these genes were mainly expressed in epithelial cells (Fig. 2C). Moreover, the distribution of the risk score was similar within this cell type (Fig. 2D).

Epithelial cells were then isolated and classified into Epi_risk_high and Epi_risk_low groups based on the median risk score (Supplementary Table 4). KEGG pathway enrichment analysis revealed that differentially expressed genes between the high- and low-risk groups were considerably enriched in cellular senescence, cell cycle, and p53 signaling pathways (Fig. 2E, Supplementary Table 5). These findings suggest that the 16 MRGs drive BCa progression by influencing these pathways. The cellular senescence pathway score was significantly elevated in the Epi_risk_high group, indicating a marked activation of cellular senescence in high-risk patients (Fig. 2F). In summary, the 16 MRGs likely contribute to BCa progression primarily via the regulation of cell cycle, p53 signaling pathway, and particularly cellular senescence.

### 3.3. DARS2 is a key regulator of BCa progression

**Figure 3:**
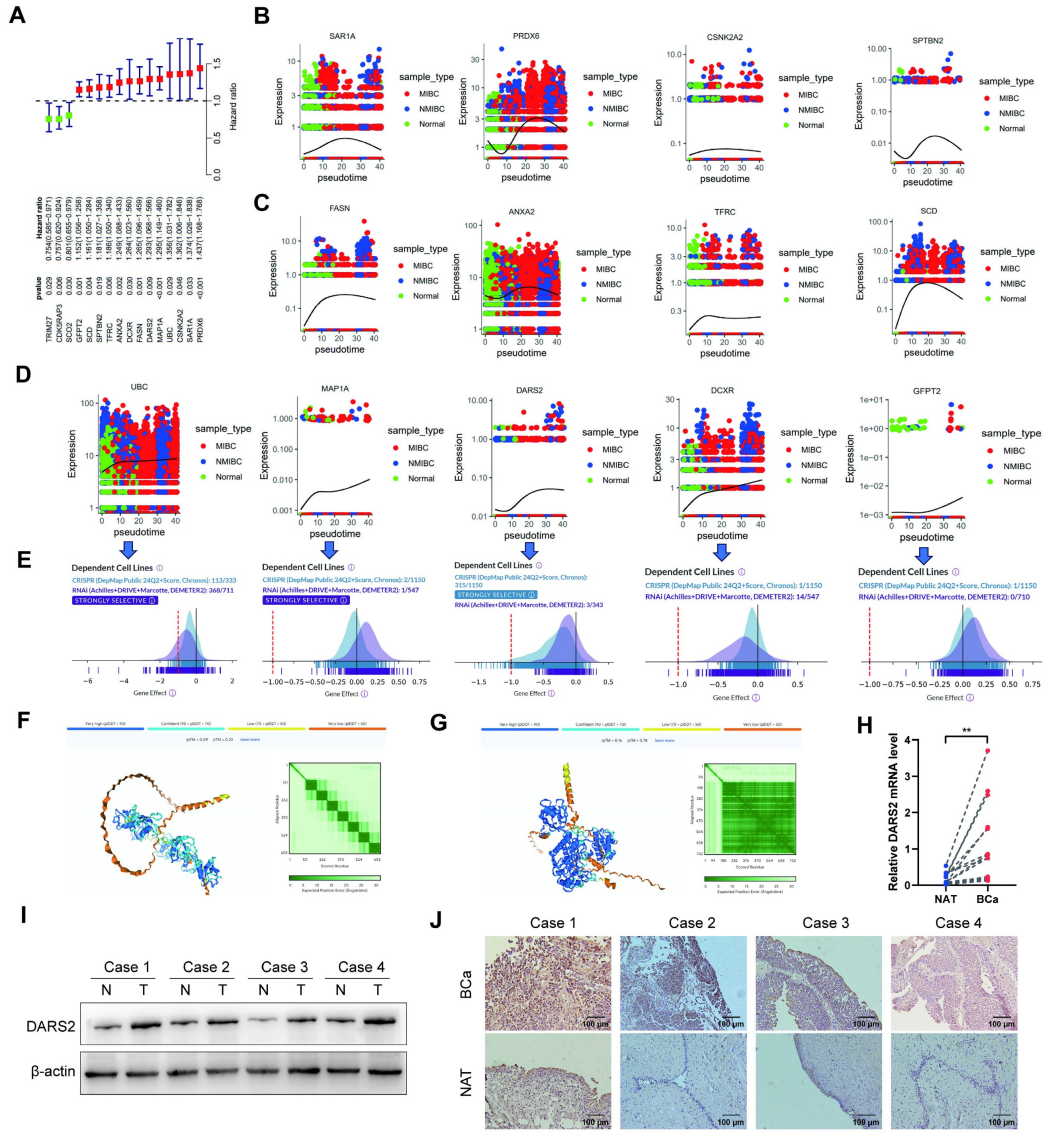
** DARS2 is identified as a key regulator in BCa progression.** (A) Univariate Cox regression identified 13 MRGs (HR >1) linked to poor prognosis. (B-D) Monocle3 pseudotime analysis revealed a progressive upregulation of UBC, MAP1A, DARS2, DCXR, and GFPT2 during the transition from normal epithelial cells to NMIBC and MIBC. (E) DepMap analysis revealed that the deletion of UBC, DARS2, and DCXR significantly inhibited tumor cell proliferation, with DARS2 displaying high dependency in 315 of 1,150 cell lines. (F-G) Alphafold3 predicted stronger interaction potential between DARS2 (G) and PINK1 when compared to UBC (F). (H-I) RT-qPCR and western blotting showed elevated DARS2 mRNA and protein levels in the BCa tissues when compared to that in the adjacent normal tissues. (J) IHC confirmed a higher DARS2 expression in the BCa tissues when compared to that in the adjacent normal tissues.

Univariate Cox regression analysis was performed to identify the most critical genes among the 16 MRGs. The findings revealed that 13 genes (HR > 1) were significantly associated with poor prognosis, underscoring their potential roles in tumor progression (Fig. 3A). Furthermore, pseudotime analysis proved that UBC, MAP1A, DARS2, DCXR, and GFPT2 were progressively upregulated during the transition from normal epithelial cells to non-muscle-invasive bladder cancer (NMIBC)- and muscle-invasive bladder cancer (MIBC)-associated cells (Fig. 3B-D). This gradual increase suggests their involvement in driving the malignancy of BCa.

DepMap demonstrated that the deletion of UBC, DARS2, and DCXR significantly inhibited tumor cell proliferation, asserting their roles in maintaining tumor growth^26^. DARS2 exhibited dependency in 315 of 1,150 cell lines, implying its importance across several cancer contexts (Fig. 3E). Also, protein interaction predictions revealed that DARS2 exhibited a stronger interaction potential with PINK1 than UBC, signifying its direct and functional role in mitophagy regulation (Fig. 3F-G). Based on its critical involvement in poor prognosis, muscle invasion, and malignant proliferation in BCa, DARS2 was identified as a candidate gene for further analyses.

In line with these observations, DARS2 expression was significantly elevated in BCa tissues at both mRNA and protein levels, confirming its role in the disease process (Fig. 3H-I). IHC staining confirmed its markedly higher expression in BCa tissues than in adjacent normal tissues, visually validating its tumor-specific up-regulation (Fig. 3J).

Collectively, these results show that DARS2 is a key regulator of BCa progression, potentially via its involvement in mitophagy and tumor cell survival.

### 3.4. DARS2 inhibits cellular senescence and promotes cell proliferation

**Figure 4:**
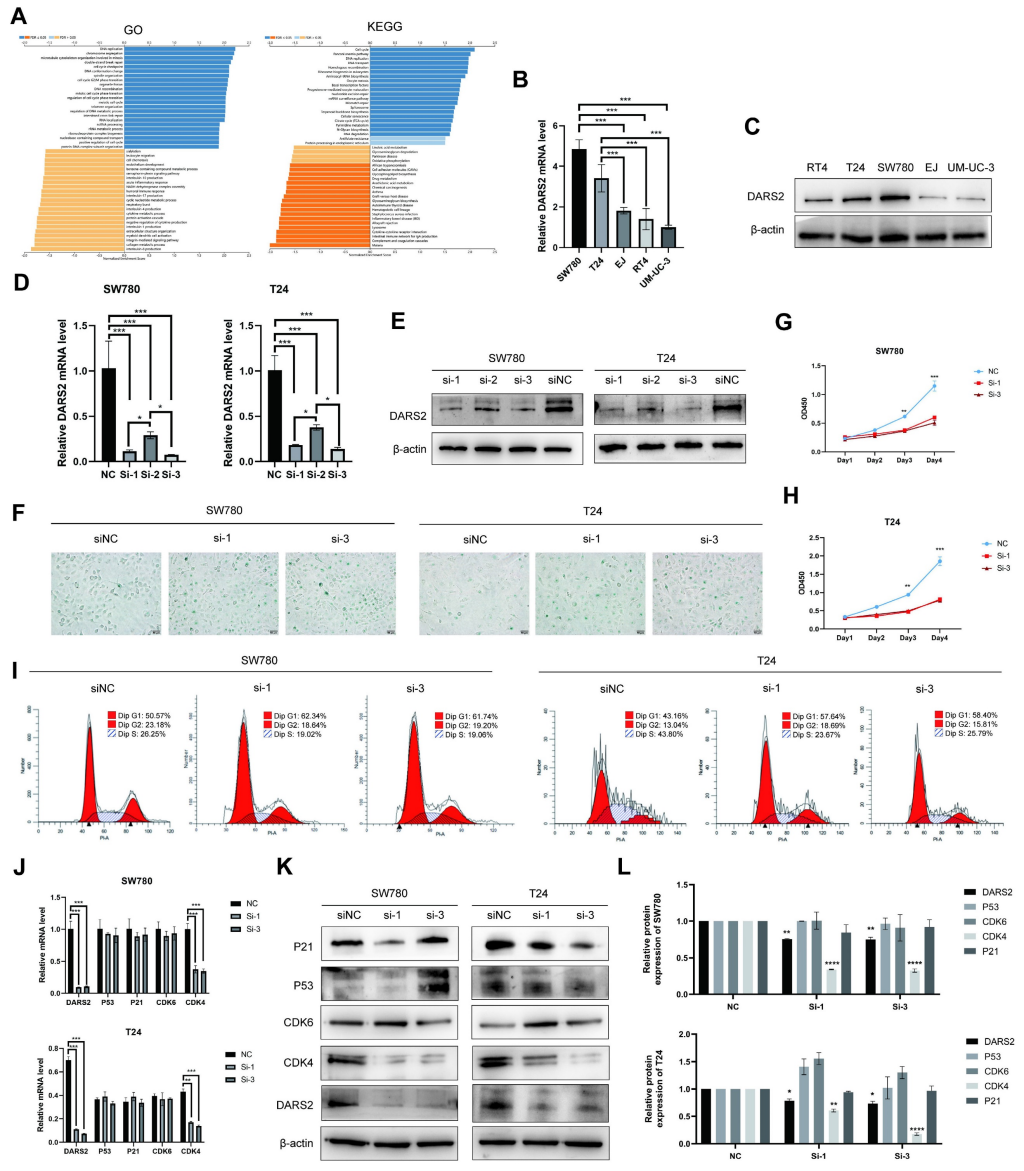
** DARS2 inhibits cellular senescence and promotes cell proliferation.** (A) GO and KEGG enrichment analyses of DARS2 in the TCGA_BLCA dataset. (B-C) The analysis of DARS2 mRNA and protein expressions in different BCa cell lines. (D-E) The verification of DARS2 knockdown by siRNA. (F) SA-β-gal staining exhibiting the effect of DARS2 knockdown on cellular senescence. (G-H) CCK8 assay showing changes in the proliferation capacity of SW780 (G) and T24 (H) cells after DARS2 knockdown. (I) Cell cycle distribution in the G1, S, and G2 phases was detected by flow cytometry and visualized using FlowJo_v10.8.1 software. (J-L) The analyses of CDK4, CDK6, p53, and p21 levels following DARS2 knockdown by siRNA.

To ascertain the biological function of DARS2 in BCa, GO and KEGG enrichment analyses were performed using the TCGA-BLCA dataset (Fig. 4A). DARS2 was chiefly enriched in pathways related to cellular senescence and cell cycle regulation, which agreed with the findings of scRNA cell analysis. DARS2 expression was evaluated in five BCa cell lines, and siRNA was used to knock down DARS2 expression in T24 and SW780 cells, which exhibited the highest DARS2 expressions (Fig. 4B-E). SA-β-gal staining was considerably increased after DARS2 knockdown, which indicated that cellular senescence was induced in BCa cells (Fig. 4F).

To further investigate the impact of DARS2 knockdown, its effects on cell proliferation and cell cycle progression were determined (Fig. 4G-I). BCa cell proliferation was significantly inhibited, and cells were arrested in the G1 phase, with a reduced number progressing to the S and G2 phases. In addition, DARS2 augmented the migration and invasion capabilities of BCa cells (Supplementary Fig. 1A). The ability of eukaryotic cells to transition from the G1 phase to the S and G2 phases is primarily regulated by G1 phase checkpoints and DNA damage checkpoints such as CDK4, CDK6, p53, and p21^27^, ^28^. CDK4 expression was reduced after DARS2 knockdown, whereas CDK6, p53, and p21 levels remained unaltered (Fig. 4J-L). These results demonstrate that DARS2 promotes G1-to-S phase transition by upregulating CDK4, thereby inhibiting cellular senescence and enhancing cell proliferation in BCa.

### 3.5. DARS2 promotes PINK1-mediated mitophagy in BCa cells

The role of DARS2 in mitophagy remained unclear. GSEA analysis of the GSE13507 dataset showed that it was significantly enriched in mitophagy-related pathways (Supplementary Fig. 1B). Moreover, correlation analysis established that DARS2 was interacted with multiple MRGs, especially PINK1 (Supplementary Fig. 1C). Notably, PINK1 protein expression was increased when DARS2 was upregulated, although no significant regulation was observed both PRKN expression and PINK1 mRNA levels (Supplementary Fig. 1D, E). Based on the known biological functions of DARS2, it was hypothesized to influence the mitochondrial synthesis of PINK1^23^. Stable DARS2-knockdown BCa cells were established, followed by the isolation of mitochondrial proteins (Supplementary Fig. 2A, B). As expected, DARS2 knockdown significantly reduced mitochondrial PINK1 protein levels (Supplementary Fig. 2C).

**Figure 5:**
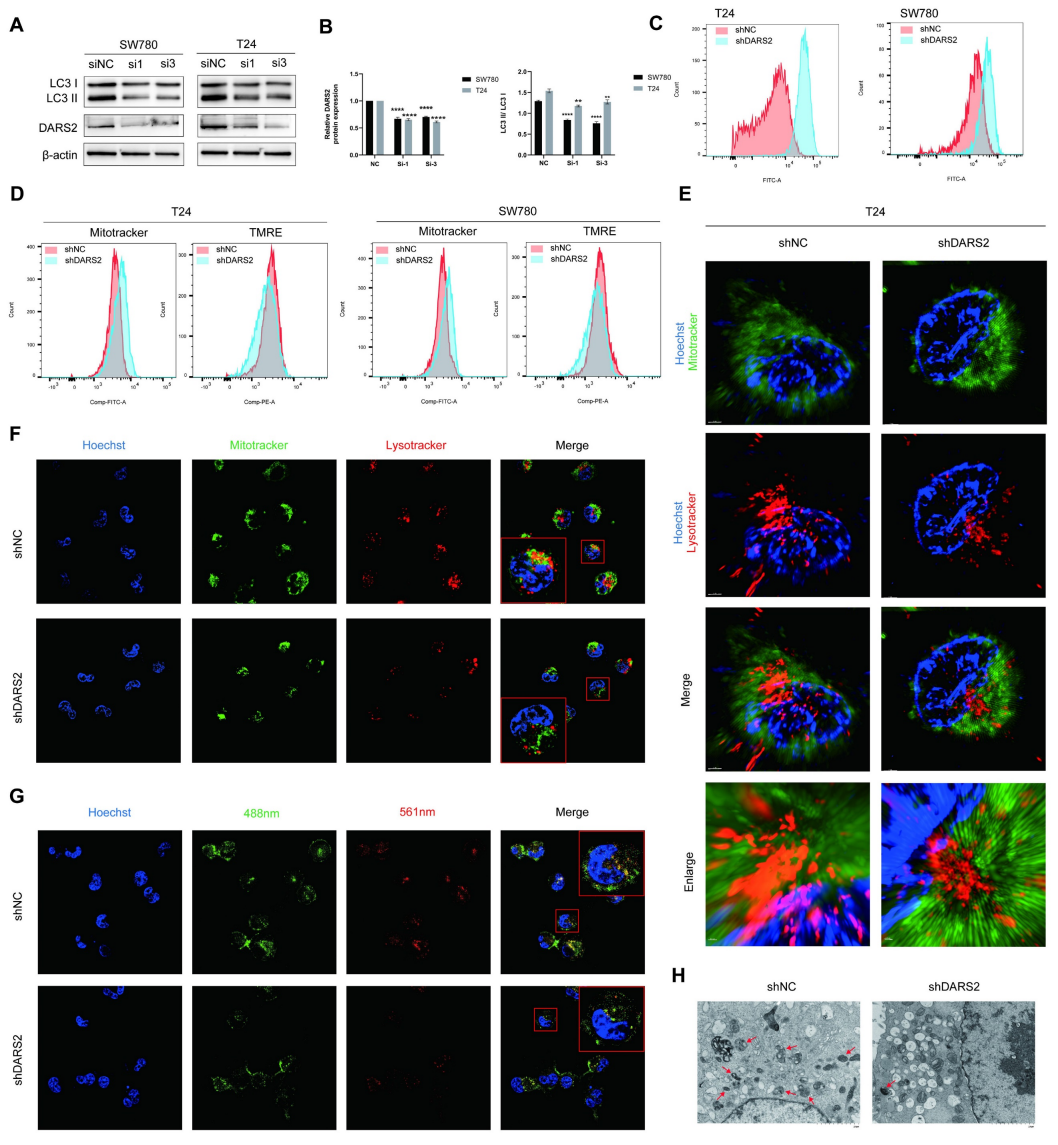
** DARS2 promotes mitophagy in BCa cells.** (A-B) Western blotting displayed decreased LC3II/I levels after DARS2 knockdown. (C) Flow cytometry revealed increased ROS levels in DARS2-knockdown cells. (D) MitoTracker-green and TMRE staining showed increased mitochondrial numbers and reduced mitochondrial membrane potential, respectively, in DARS2-knockdown cells. (E) Super-resolution microscopy revealed reduced mitochondrial-lysosomal colocalization in DARS2-knockdown T24 cells. (F) Confocal microscopy confirmed reduced mitochondrial-lysosomal colocalization in DARS2-knockdown SW780 cells, with MitoTracker-green for the mitochondria, Lysotracker-red for lysosomes, and Hoechst for nuclei. (G) Mt-Keima fluorescence intensity revealed reduced colocalization of mitochondria (488 nm) and lysosomes (561 nm) in DARS2-knockdown SW780 cells. (H) Transmission electron microscopy revealed decreased mitophagy and increased vacuolization in DARS2-knockdown T24 cells.

To determine the effect of DARS2 on mitophagy, LC3II/I levels were analyzed and found to be decreased after DARS2 knockdown (Fig. 5A, B). Considering the intricate association between ROS and mitophagy, ROS levels were measured before determining mitophagy^29^, ^30^. ROS levels were significantly elevated in DARS2-knockdown cells, implying impaired mitochondrial quality control (Fig. 5C). Mitotracker and TMRE staining showed that although the total number of mitochondria was increased, the proportion of functional mitochondria with an intact membrane potential was decreased (Fig. 5D). These observations suggest that DARS2 knockdown impairs the clearance of damaged mitochondria, causing dysfunctional mitochondria to accumulate.

Super-resolution and confocal microscopy using Mitotracker-green and Lysotracker-red established that the colocalization of mitochondria and lysosomes was reduced in DARS2-knockdown cells, implying decreased mitophagy (Fig. 5E, F). Furthermore, mt-Keima assays showed reduced colocalization of mitochondria (neutral pH, green) and lysosomes (acidic pH, red), which agreed with impaired mitophagy (Fig. 5G). In addition, transmission electron microscopy revealed mitochondrial vacuolization and swelling, signifying decreased levels of mitophagy (Fig. 5H). These findings assert that DARS2 enhances PINK1-mediated mitophagy by regulating PINK1 mitochondrial synthesis and contributes to mitochondrial quality control in BCa cells.

### 3.6. DARS2 inhibits cellular senescence and promotes tumor growth by enhancing PINK1-mediated mitophagy

**Figure 6:**
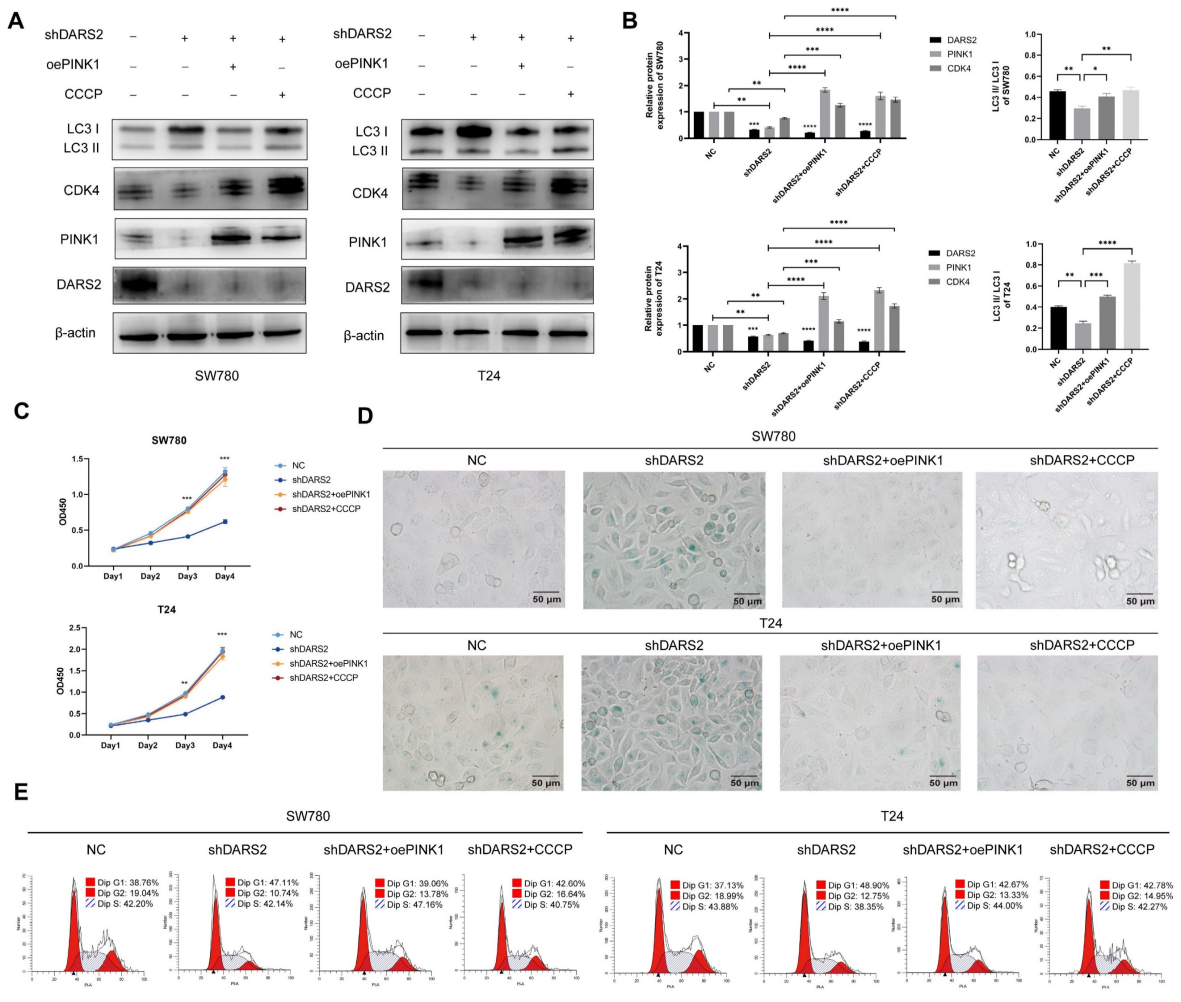
** DARS2 inhibits cellular senescence by promoting PINK1-mediated mitophagy.** (A-B) Western blotting analysis of the protein expression following stable DARS2 knockdown and transfection with overexpressed PINK1 plasmids and CCCP treatment (10 μM CCCP for 24 h). (C) Cell proliferation was evaluated by the CCK8 assay. (D) Cellular senescence was assessed by SA-β-gal staining. (E) Cell cycle distribution in the G1, S, and G2 phases was detected by flow cytometry and visualized by using FlowJo_v10.8.1 software.

To further determine the association between DARS2-regulated mitophagy and cellular senescence, PINK1 was overexpressed and DARS2-knockdown BCa cells were treated with CCCP. It is a protonophore that augments the permeability of the mitochondrial inner membrane to H^+^, leading to a loss of membrane potential and accelerating mitochondrial depolarization and mitophagy^31^. PINK1 overexpression and CCCP treatment significantly increased autophagy levels and partially restored the decreased CDK4 expression attributed to DARS2 knockdown (Fig. 6A, B).

In addition, PINK1 overexpression and CCCP treatment significantly decreased SA-β-gal positivity and enhanced cell proliferation compared with the DARS2-knockdown group (Fig. 6C, D). More pertinently, this treatment alleviated the G1 phase arrest caused by DARS2 knockdown (Fig. 6E). These results signify that DARS2 promotes CDK4 expression and facilitates the G1-to-S phase transition by enhancing PINK1-mediated mitophagy, inhibiting cellular senescence and stimulating cell proliferation.

**Figure 7:**
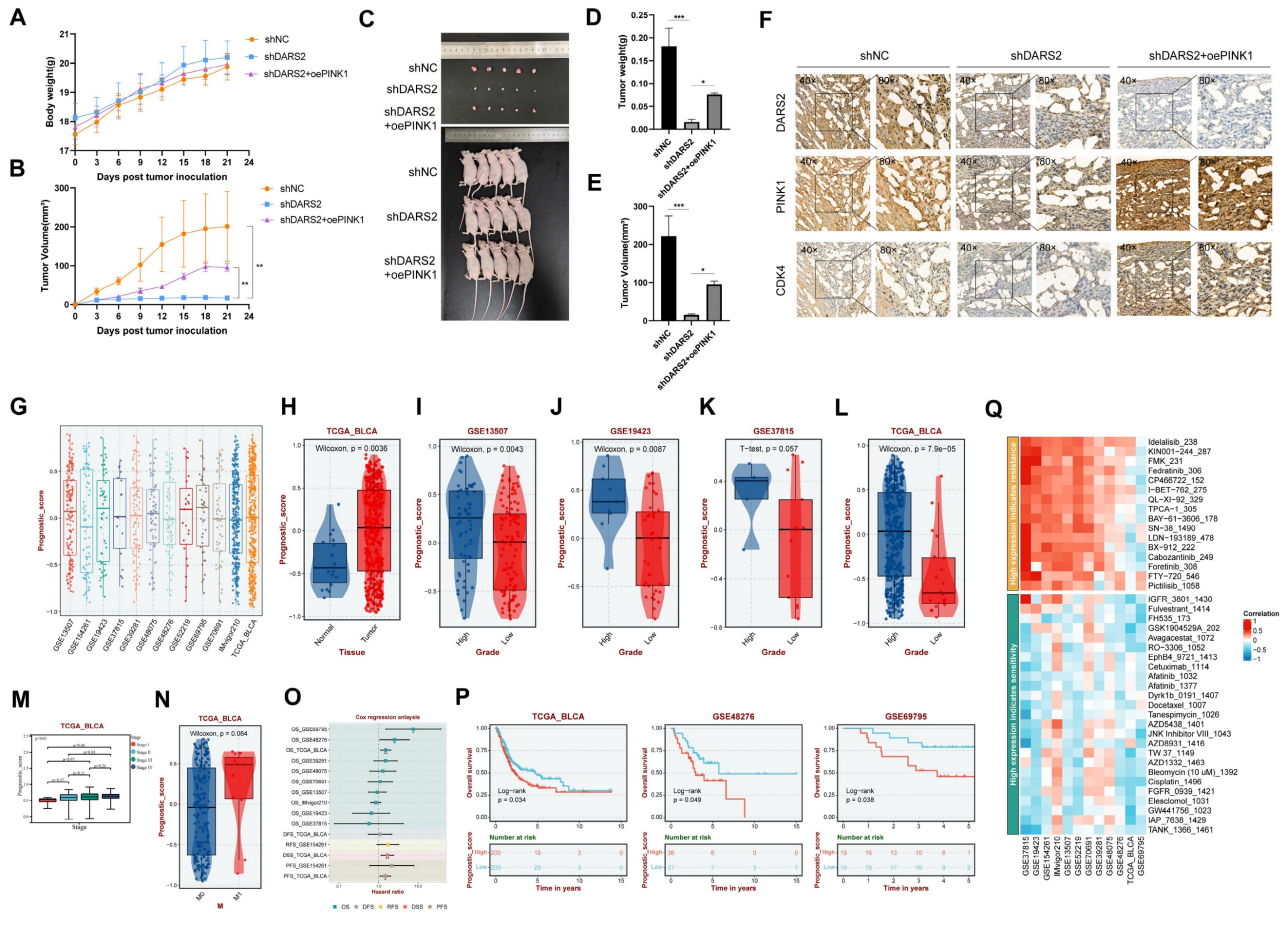
** Clinical significance of the DARS2-PINK1-CDK4 axis.** (A-B) Nude mice were subcutaneously injected with BCa cells and monitored for body weight (A) and tumor volume (B) from day 0 to day 21. (C) Gross images of nude mice and tumors at the endpoint. (D-E) Tumor weight (D) and volume (E) were measured after euthanasia. (F) Immunohistochemical analysis revealed the expressions of DARS2, PINK1, and CDK4 in subcutaneous tumors. (G) The DARS2-PINK1-CDK4 axis expressions were calculated across BCa cohorts to generate a Prognostic Score. (H-N) The expressions of the axis varied significantly across different tissue types, tumor grades, stages, and metastasis statuses in multiple datasets. (O) Multivariate regression analysis evaluated the prognostic value of the axis across databases. (P) Survival analysis showed the impact of the axis on overall survival in multiple datasets. (Q) Sensitivity analysis revealed the axis's response to various drug treatments.

To evaluate the impacts of DARS2 on tumor growth *in vivo*, subcutaneous tumor xenograft experiments were conducted using nude mice (Fig. 7A-C). DARS2 knockdown significantly reduced tumor size and weight, an effect partially reversed by PINK1 overexpression (Fig. 7D, E). In addition, IHC analysis of the tumor tissues confirmed that DARS2 knockdown considerably reduced PINK1 and CDK4 expressions, which were partially restored upon PINK1 overexpression, consistent with our earlier findings (Fig. 7F). These observations assert that DARS2 knockdown exerts an antitumor effect by promoting PINK1-dependent mitophagy.

The clinical significance of the DARS2-PINK1-CDK4 axis was explored by applying the ssGSEA algorithm to determine the overall expressions of this axis across multiple BCa cohorts, generating a prognostic score (Fig. 7G). This axis was highly expressed in BCa and was significantly positively correlated with higher tumor grade, stage, and distant metastasis across multiple datasets (Fig. 7H-N). Furthermore, it was significantly linked to poor overall survival outcomes and suboptimal therapeutic responses to various drugs (Fig. 7O-Q, Supplementary Table 6). Hence, targeting the DARS2-PINK1-CDK4 axis holds promise for improving the prognosis of patients with BCa.

## 4. Discussion

BCa is one of the most common malignant tumors affecting the urinary system worldwide. The disease's pathological types and progression mechanisms are complex^3^, ^32^. Patients often face challenges such as high recurrence rates, chemotherapy resistance, and poor prognosis^3^, ^33^, ^34^. Therefore, determining the molecular mechanisms underlying BCa and identifying novel therapeutic targets is of immense clinical importance. The role of cellular senescence in cancer has recently garnered considerable research attention^35^, ^36^. Cellular senescence is marked by irreversible cell cycle arrest and serves as a stress response that inhibits cell proliferation, preventing the malignant spread of cancer cells^37^. Nonetheless, senescent cells secrete proinflammatory factors and matrix-degrading enzymes, collectively known as SASPs, activating the tumor microenvironment and promoting tumor progression^13^, ^38^. This dual nature of cellular senescence emphasizes its complex role in cancer development. Comprehending the molecular basis of this process in BCa is crucial for gaining insights into tumorigenesis and developing new treatment strategies.

Mitophagy is responsible for clearing damaged mitochondria and maintaining the health of the organelle. This process is involved in energy metabolism and the reduction of ROS accumulation within cells^18^, ^39^. The role of mitophagy in cancer is complex. On the one hand, it suppresses tumorigenesis by eliminating damaged mitochondria and reducing ROS levels^40^. On the other hand, excessive mitophagy may provide energy for cancer cells and promote tumor progression^40^, ^41^. Because of this duality, mitophagy is a controversial and challenging cancer research area.

In this study, several machine learning algorithms were used to develop a prognostic model based on MRGs, identifying 16 genes closely linked to BCa prognosis, with DARS2 emerging as a key gene. DARS2 encodes mitochondrial aspartyl-tRNA synthetase, a vital enzyme in mitochondrial protein synthesis^23^. Although previous studies have implicated DARS2 in liver and lung cancer progression, its role in BCa has not been completely elucidated^24^, ^25^. *In vitro* experiments with BCa cell lines indicated that silencing DARS2 substantially inhibited cell proliferation and promoted cellular senescence. Specifically, DARS2 knockdown downregulated CDK4, induced cell cycle arrest in the G1 phase, increased SA-β-gal staining, and reduced cell proliferation. These findings imply that DARS2 is an imperative regulatory factor in BCa cells.

Subsequently, whether DARS2 regulates cellular senescence in BCa cells via a mitophagy-dependent pathway was examined. PINK1, a crucial regulator of the mitophagy pathway, accumulates on the outer mitochondrial membrane in response to damage to this organelle and recruits Parkin for the autophagic degradation of damaged mitochondria^42^. This was the first study to demonstrate the association between DARS2 and PINK1. Our findings revealed that DARS2 knockdown significantly down-regulated the PINK1 protein expression in BCa cells, although the PINK1 mRNA levels were not significantly altered. Isolation of mitochondrial proteins established that DARS2 knockdown inhibited mitochondrial synthesis of PINK1, which explained the phenomenon above. Furthermore, immunofluorescence and confocal microscopy showed that DARS2 knockdown significantly decreased the colocalization of mitochondria and lysosomes, implying reduced mitophagy. In addition, transmission electron microscopy revealed enhanced vacuolization and mitochondrial swelling in DARS2-knockdown cells, which are typical signs of impaired mitophagy. These findings together suggested that DARS2 elevated the PINK1 protein levels by promoting its mitochondrial synthesis, thereby enhancing PINK1-mediated mitophagy.

Moreover, in BCa cells, DARS2 knockdown significantly increased the levels of ROS, a major inducer of mitophagy. Under normal conditions, mitophagy effectively eliminates damaged mitochondria and maintains stable intracellular ROS levels^30^. However, DARS2 knockdown resulted in mitochondrial damage accumulation, further accentuating the ROS levels, which could be a result of decreased mitophagy and a cause of increased cellular senescence. Overexpression of PINK1 or treatment with the autophagy inducer CCCP partially restored the reduction in mitophagy and the increase in cellular senescence caused by DARS2 knockdown. These observations confirm that DARS2 regulates BCa cellular senescence via PINK1-mediated mitophagy.

The relationship between cellular senescence and proliferation in BCa progression was further investigated. It is reported that cellular senescence is closely related to proliferation as well as cell cycle arrest^43^-^46^. However, the relationship between cellular senescence and proliferation in BCa has not yet been fully elucidated. In this study, DARS2 was found to inhibit cellular senescence and promote cell proliferation. However, the causal relationship between cellular senescence and proliferation still requires further confirmation.

Previous research on DARS2 has focused on its role in neurological diseases^23^. This study is the first to examine its involvement in BCa, revealing its critical role in regulating the cell cycle, inhibiting cellular senescence, and promoting cell proliferation. According to the proposed molecular mechanism, DARS2 suppresses cellular senescence and stimulates BCa progression via PINK1-mediated mitophagy, offering new insights into the roles of DARS2 and mitophagy in BCa. Moreover, this study clarified the clinical relevance of the DARS2-PINK1-CDK4 axis in BCa. Bioinformatics analysis proved that the elevated expression of this axis was considerably linked to poor prognosis in patients with BCa, suggesting that targeting this axis is a promising therapeutic approach, especially for patients who respond poorly to conventional chemotherapy or immunotherapy.

Despite elucidating the importance of DARS2 in BCa, some limitations should be addressed in our present study. Firstly, it remains unclear whether DARS2 influences mitophagy through other pathways. Furthermore, the variability of the role of DARS2 across different BCa subtypes should be considered. In addition, the potential targets of DARS2 and PINK1 for precise BCa treatment may be explored.

## 5. Conclusions

In this study, a prognostic model was constructed by screening 16 MRGs, which exhibited a strong predictive value for the prognosis of patients with BCa. Of these genes, DARS2 was a key regulator and was found to be upregulated in BCa tissues. Silencing of DARS2 markedly induced cellular senescence and inhibited the proliferation of BCa cells via PINK1-mediated mitophagy. Mechanistically, the mitophagy inhibition caused by DARS2 silencing led to a decrease in CDK4 levels. This reduction resulted in cell cycle arrest in the G1 phase and limited the transition of cells into the S and G2 phases. In conclusion, DARS2 demonstrates significant predictive effects on BCa progression and may serve as a promising therapeutic target in the future.

## Supplementary Material

Supplementary figures.

Supplementary tables.

## Abbreviations

BCa: Bladder cancer
NMIBC: Nonmuscle invasive bladder cancer
MIBC: Muscle invasive bladder cancer
OXPHOS: Oxidative phosphorylation
ROS: Reactive oxygen species
DARS2: Aspartyl-tRNA synthetase 2
SASPs: Senescence-associated secretory phenotypes
MRGs: Mitophagy-related genes
GSEA: Gene set enrichment analysis
DEGs: Differentially expressed genes
GO: Gene Ontology
KEGG: Kyoto Encyclopedia of Genes and Genomes
TMRE: Tetramethylrhodamine ethyl ester
CCCP: Carbonyl cyanide m-chlorophenyl hydrazone
