# Supplementary material for: DARS2 Promotes Bladder Cancer Progression by Enhancing PINK1-Mediated Mitophagy
Source: Int J Biol Sci. 2025 Jan 27;21(4):1530–44. doi: 10.7150/ijbs.107632 (PMC11844284; doi:10.7150/ijbs.107632)
Supplement: Supplementary file 1 — Supplementary figures. [file ijbsv21p1530s1.pdf]

Supplementary Figure 1

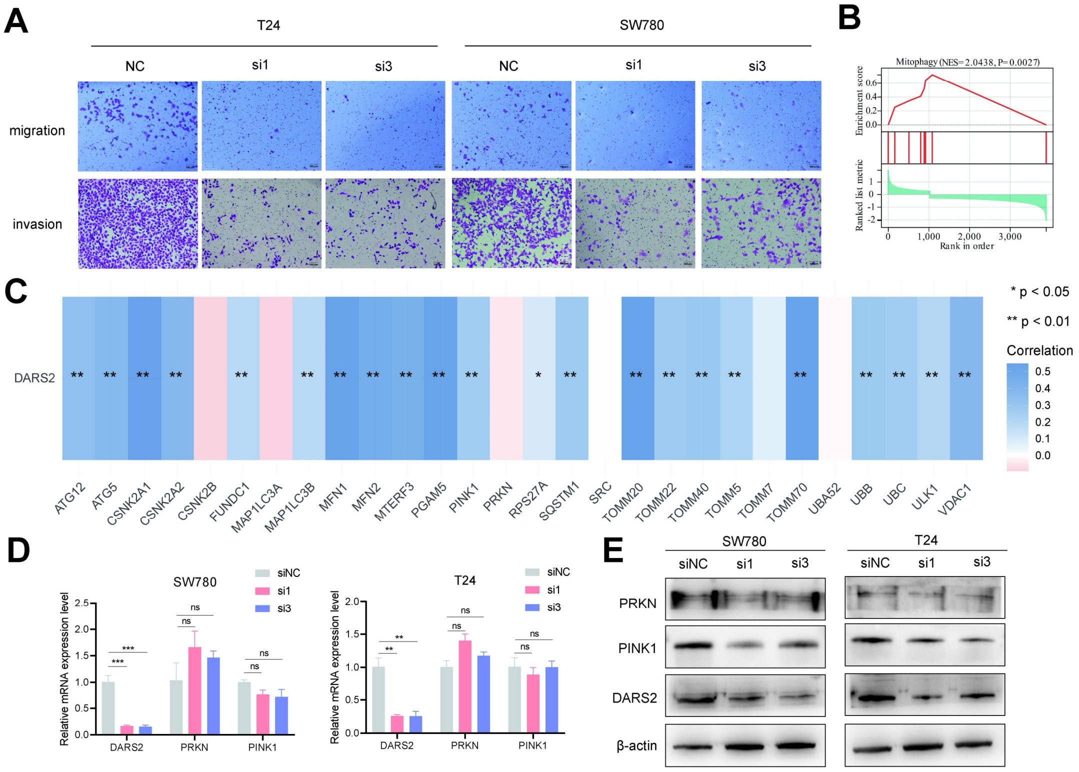

Supplementary Figure. 1. DARS2 is related to mitophagy.

(A) Transwell assay exhibited the migration as well as invasion abilities of BCa cells after DARS2 knockdown. (B) GSEA enrichment analysis of DARS2-related genes in the GSE13507 dataset. (C) Correlation analysis between DARS2 and mitophagy-related genes. (D–E) Analysis of PINK1 and PRKN mRNA (D) and protein levels (E) following DARS2 knockdown.

Supplementary Figure 2

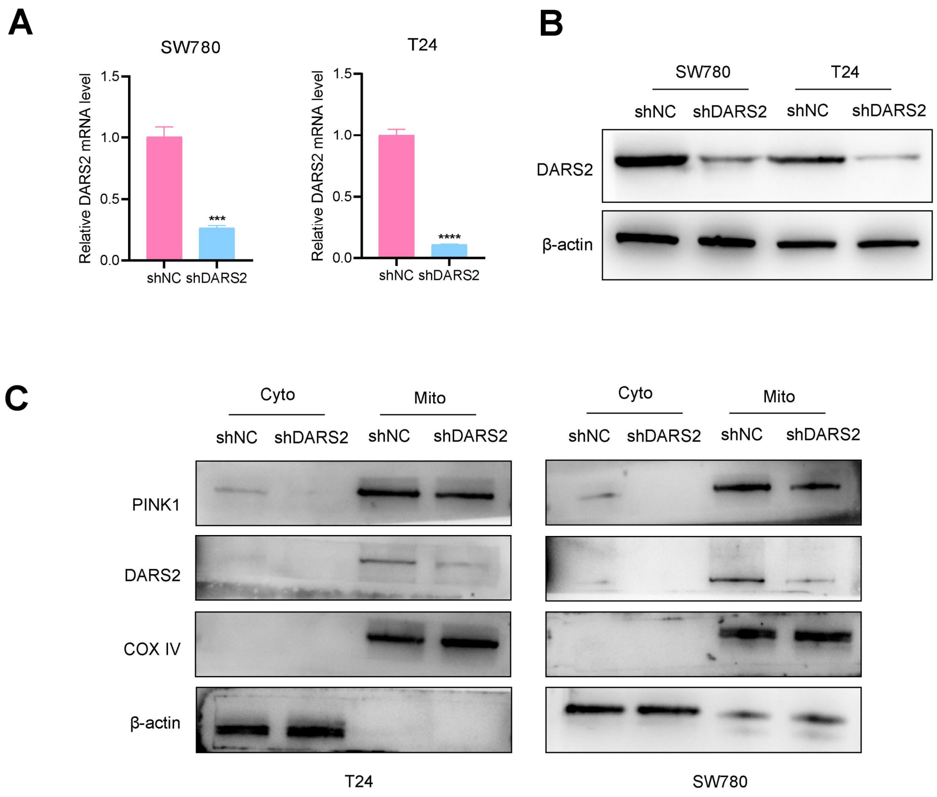

Supplementary Figure. 2. DARS2 promotes PINK1 mitochondrial synthesis.

(A–B) RT-qPCR (A) and western blotting (B) confirmed the efficiency of DARS2 knockdown. (C) Western blotting showed the protein levels of PINK1 in mitochondrial and cytoplasmic fractions following DARS2 knockdown. β-actin was used as the loading control for cytoplasmic proteins. COX IV was used as the loading control for mitochondrial proteins.
